# Supplementary material for: Exploring Pt-Impregnated CdS/TiO2 Heterostructures for CO2 Photoreduction
Source: Nanomaterials (Basel). 2024 Nov 12;14(22):1809. doi: 10.3390/nano14221809 (PMC11597567; doi:10.3390/nano14221809)
Supplement: Supplementary file 1 [file nanomaterials-14-01809-s001.zip › nanomaterials-3268568-supplementary.pdf]

Supporting Information

Article

Exploring Pt-Impregnated CdS/TiO_2 Heterostructures for CO_2 Photoreduction

Lidia García-Santos, Javier Fernández-Catalá, Ángel Berenguer-Murcia * and Diego Cazorla-Amorós

Inorganic Chemistry Department, Materials Science Institute, University of Alicante, Ap. 99, 03080 Alicante, Spain; lidia.garciasantos@ua.es (L.G.-S.); j.fernandezcatala@ua.es (J.F.-C.); cazorla@ua.es (D.C.-A.)
* Correspondence: a.berenguer@ua.es; Tel.: +34-96-590-3946

S1. Materials and Methods

S1.1 Synthesis of CdS/TiO_2 Photocatalysts

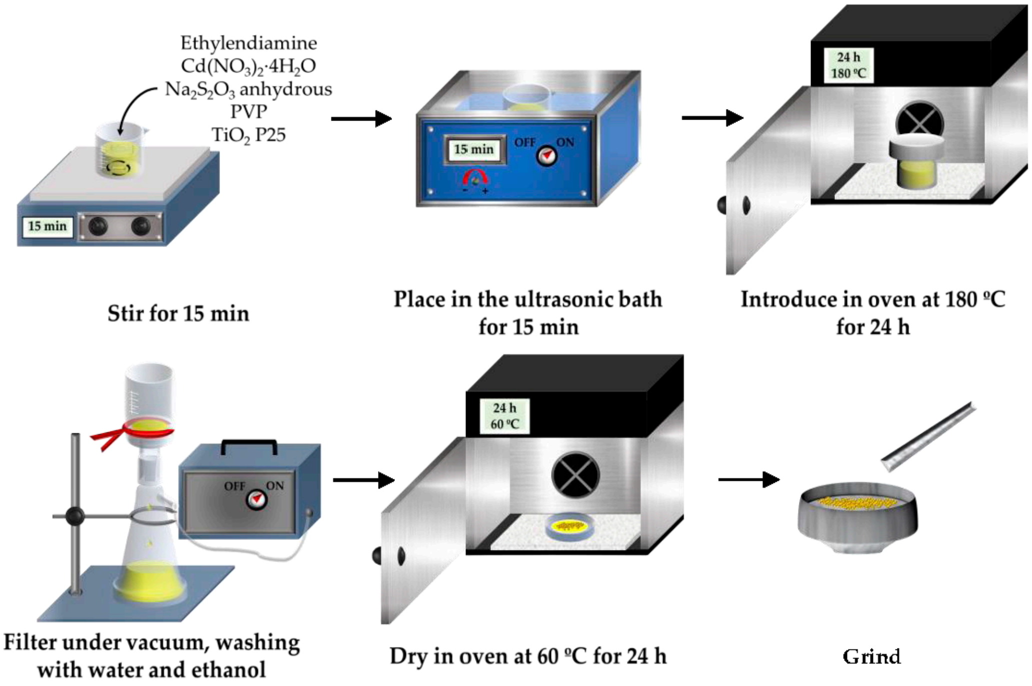

Figure S1. Scheme of the synthesis of CdS/TiO_2 photocatalysts.

S1.2 Synthesis of Pt/CdS/TiO$_2$ Photocatalysts

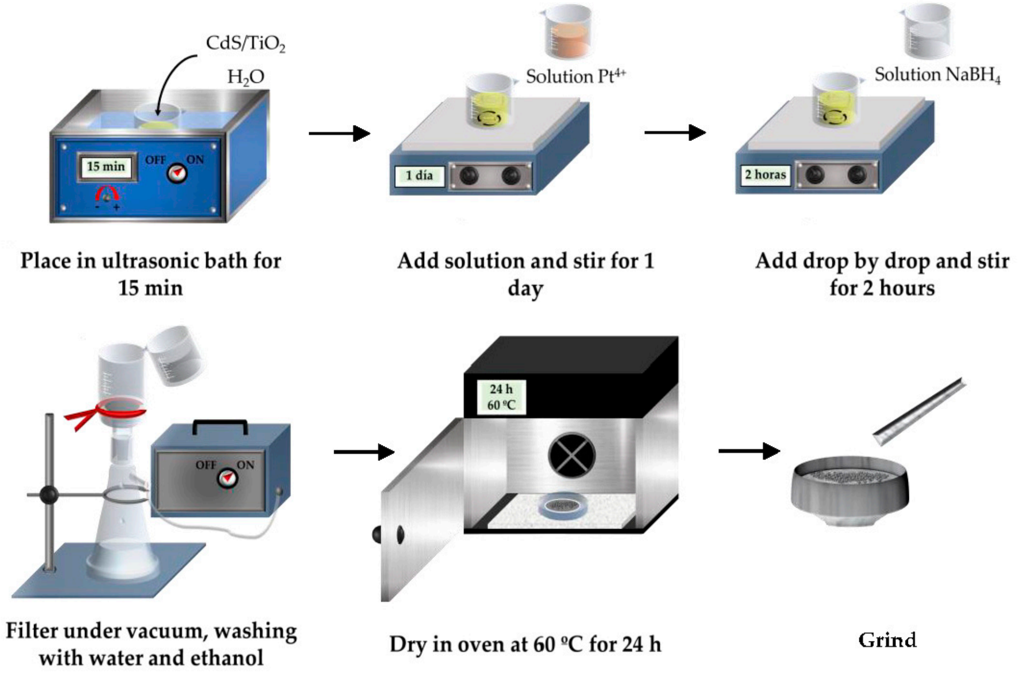

Figure S2. Scheme of the synthesis of Pt/CdS/TiO$_2$ photocatalysts.

S2. Results and Discussion

S2.1 Characterization Results

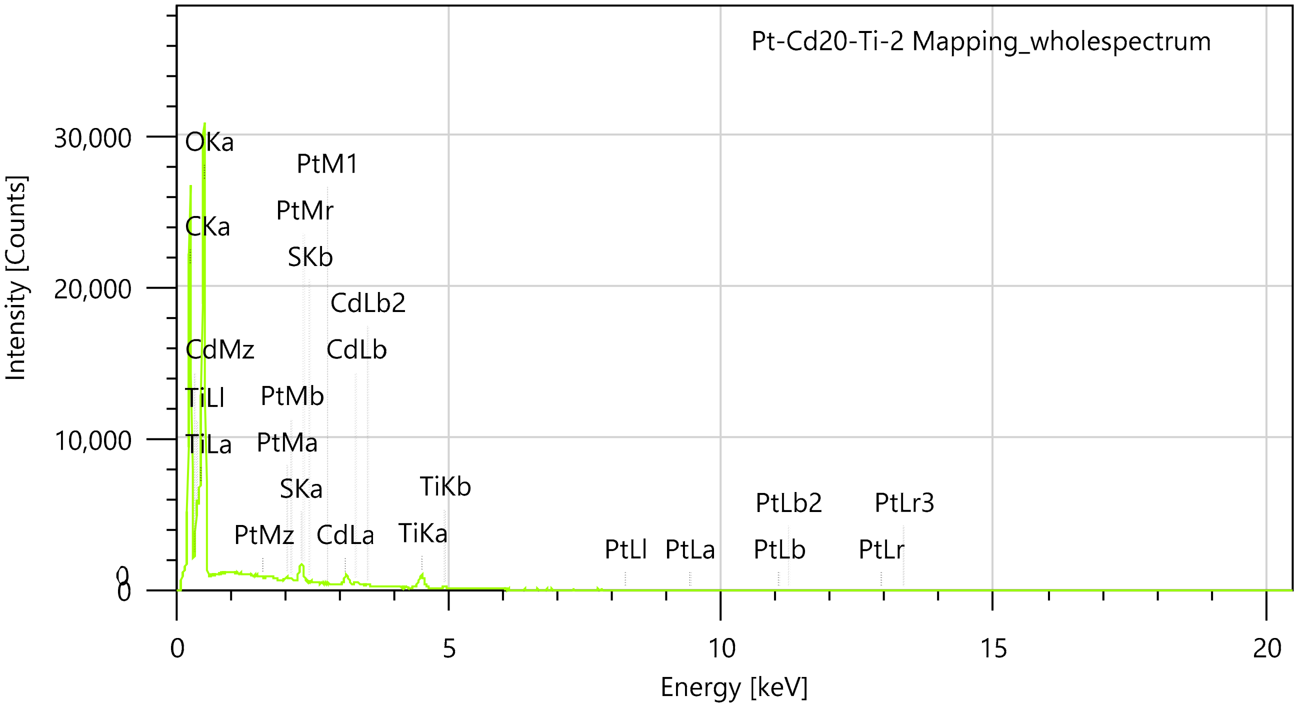

Figure S3. EDS spectra of the Pt/Cd(20)-Ti sample.

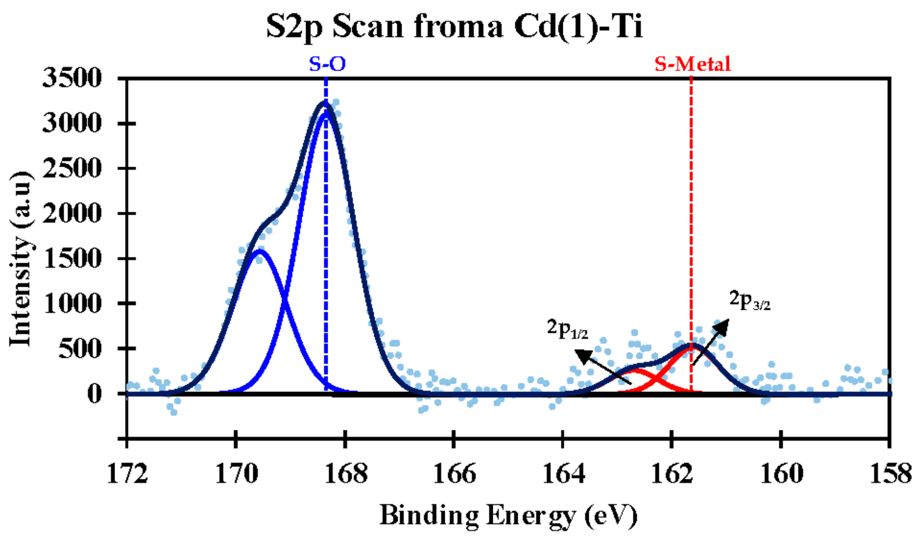

Figure S4. XPS spectra of the S2p scan from the Cd(1)-Ti sample.

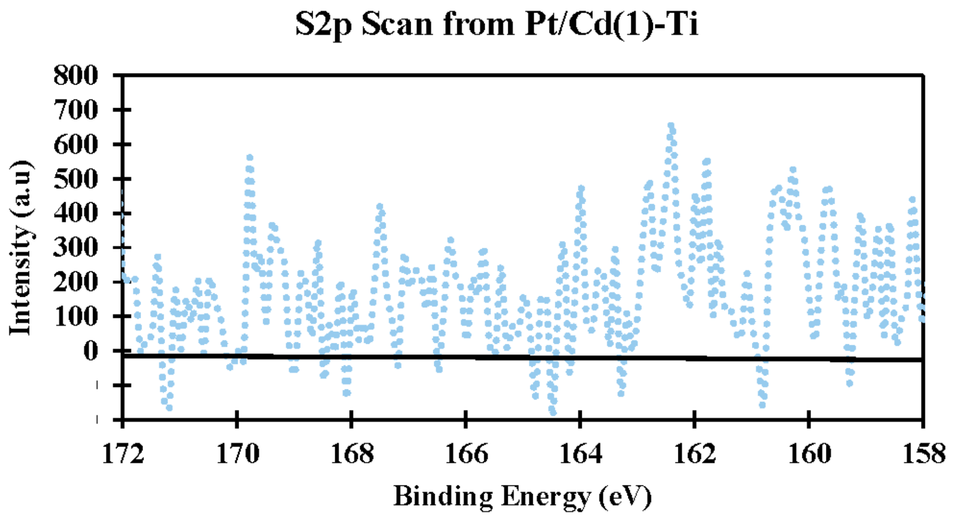

Figure S5. XPS spectra of the S2p scan from the Pt/Cd(1)-Ti sample.

To perform the calculations to quantify the amounts of generated product, we must know the relationship between the obtained peak area and methane concentration in ppm. For this purpose, the equipment was calibrated using a CH_4 gas cylinder with a calibrated concentration of 1901 ppm in He, which corresponds to 2270.9 area units of methane on the chromatograph. As an example, we will calculate the concentration of CH_4 for the Pt/Cd(1)-Ti test:

$$3234.20\ unit\ per\ area\ CH_4 \cdot \frac{1901\ ppm\ CH_4}{2270.90\ unit\ per\ area\ CH_4} = 2707.34\ ppm\ CH_4$$

Knowing that 1 ppm equals 1 µL/L and that the gas flow rate is 5 mL/min, we can calculate the µL of CH_4 per minute as follows:

$$\frac{2707.34\ \mu L\ CH_4}{L} \cdot \frac{5 \cdot 10^{-3}\ L}{min} = 13.54\ \mu L\ CH4/min$$

Assuming that CH_4 behaves as an ideal gas at room temperature (298 K) and standard pressure (1 atm), we can calculate the µmol of CH_4 per minute using the ideal gas law P·V=n·R·T. Knowing the catalyst mass is 0.04 g, we can also determine the µmol CH_4 per gram of catalyst per hour:

$$n = \frac{1 \cdot 13.54}{0.08205 \cdot 298} = 5.54 \cdot 10^{-1}\ \mu mol\ CH_4/min$$

$$\frac{5.54 \cdot 10^{-1}\ \mu mol\ CH_4}{0.04\ g\ catalyst \cdot min} \cdot \frac{60\ min}{1\ hour} = 830.27\ \mu mol\ CH_4/g \cdot h$$

From this value, we need to subtract the amount of CH_4 produced from ethanol, which was measured in the blank run. The same procedure was followed, yielding 369.93 µmol CH_4/g·h. Therefore, the µmol CH_4/g·h exclusively from CO_2 is as follows:

$$830.27 - 369.93 = 460.34\ \mu mol\ CH_4/g \cdot h$$

Acetaldehyde was calibrated similarly to methane using a gas with a known composition of this compound injected into the gas bubbler.
